# Supplementary material for: Farey tree and devil’s staircase of frequency-locked breathers in ultrafast lasers
Source: Nat Commun. 2022 Oct 2;13:5784. doi: 10.1038/s41467-022-33525-0 (PMC9527256; doi:10.1038/s41467-022-33525-0)
Supplement: Supplementary file 1 — Supplementary Information [file 41467_2022_33525_MOESM1_ESM.pdf]

Farey tree and devil's staircase of frequency-locked breathers in ultrafast lasers: Supplementary Information

Xiuqi Wu[1], Ying Zhang[1], Junsong Peng[1,2*], Sonia Boscolo[3], Christophe Finot[4], Heping Zeng[1,5,6*]

[1]State Key Laboratory of Precision Spectroscopy, East China Normal University, Shanghai 200062, China
[2]Collaborative Innovation Center of Extreme Optics, Shanxi University, Taiyuan, Shanxi 030006, People's Republic of China
[3]Aston Institute of Photonic Technologies, Aston University, Birmingham B4 7ET, United Kingdom
[4]Laboratoire Interdisciplinaire Carnot de Bourgogne, UMR 6303 CNRS – Université de Bourgogne Franche-Comté, F-21078 Dijon Cedex, France
[5]Chongqing Institute of East China Normal University, Chongqing 401120, China
[6]Shanghai Research Center for Quantum Sciences, Shanghai 201315, China

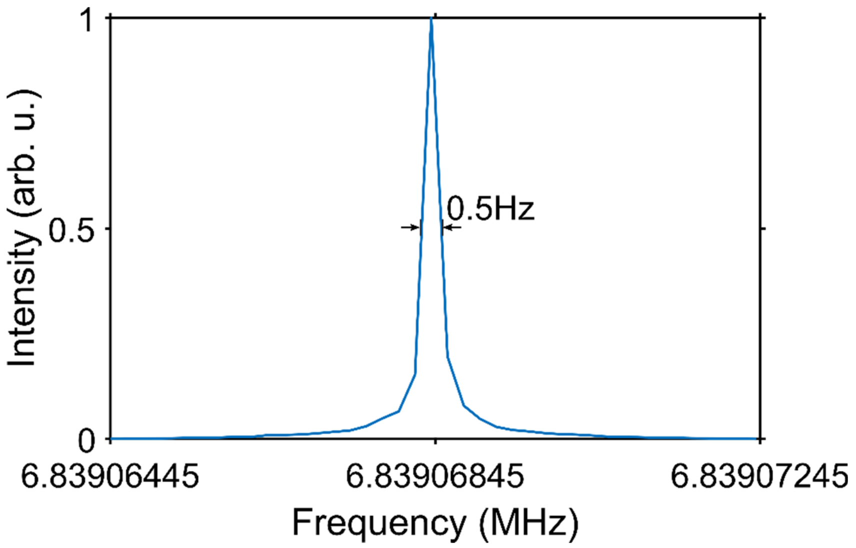

Fig. 1 Measurement of the linewidth of the breathing frequency for the 1/5 locked breather state illustrated in the main body of the manuscript. Since the ESA cannot resolve the narrow linewidth of the breathing frequency, we have used a different method for its measurement: after being passed through a filter to remove its harmonics and and the cavity repetition frequency, the breathing frequency is delivered to the oscilloscope and a long time trace is recorded (5 seconds, corresponding to a frequency resolution of 0.2 Hz). The breathing frequency's linewidth can be then obtained with a high resolution from the processing of the fast Fourier transform of the time trace.

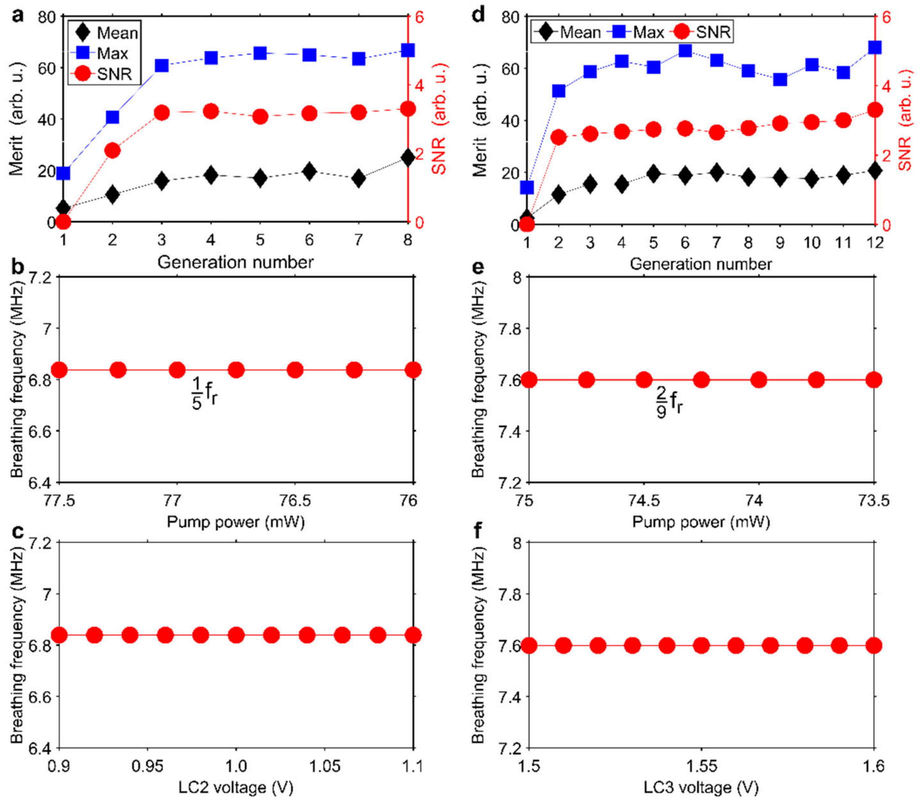

Fig. 2 EA optimisation of breather states with winding numbers of 1/5 (panels (a-c)) and 2/9 (panels (d-f)). (a, d) Evolution of the average (black diamonds) and maximum (blue squares) merit scores over successive generations, for the merit function given in Eq. (2) (Methods). Also shown is the corresponding evolution of the SNR of the breathing frequency (red circles). Persistence of the optimal state with variation of the (b, e) pump power and (c, f) polarisation (varied by changing the voltage on LC3).

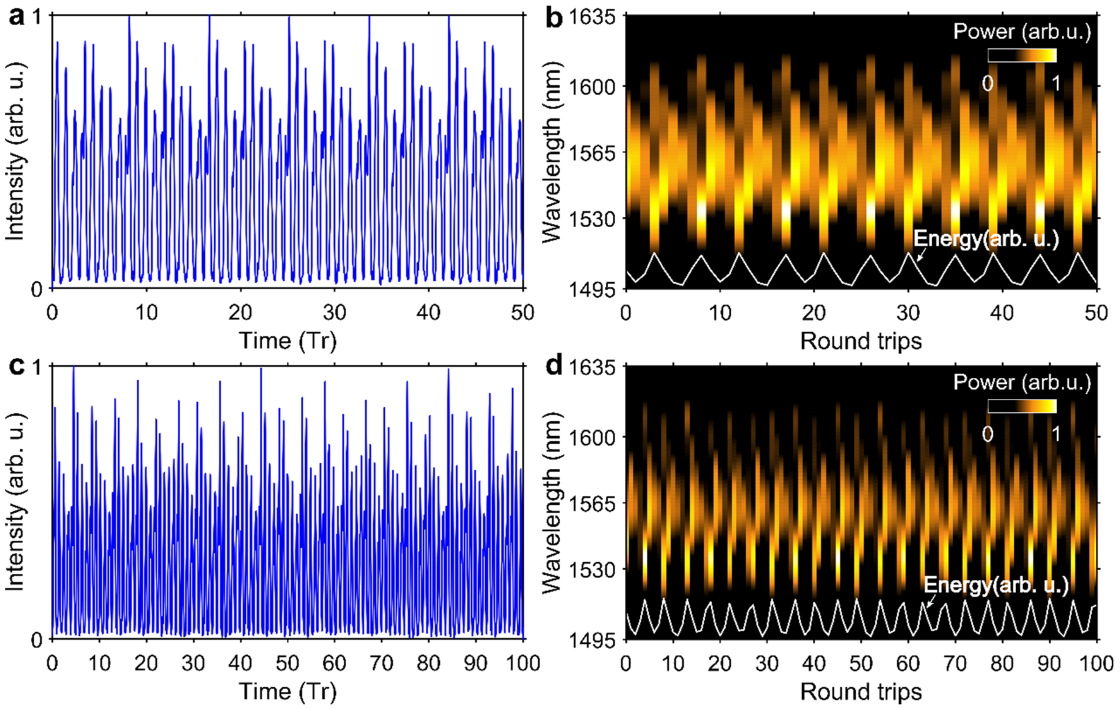

Fig. 3 Evolution dynamics of frequency-locked breather states with winding numbers of 2/9 and 9/41 (panels (a, b) and (c, d), respectively). Panels (a, c) show the photo-detected DFT output signals (T_r is the roundtrip time), and panels (b, d) are the corresponding DFT recordings of single-shot spectra. The white curves in (b, d) represent the energy evolutions.

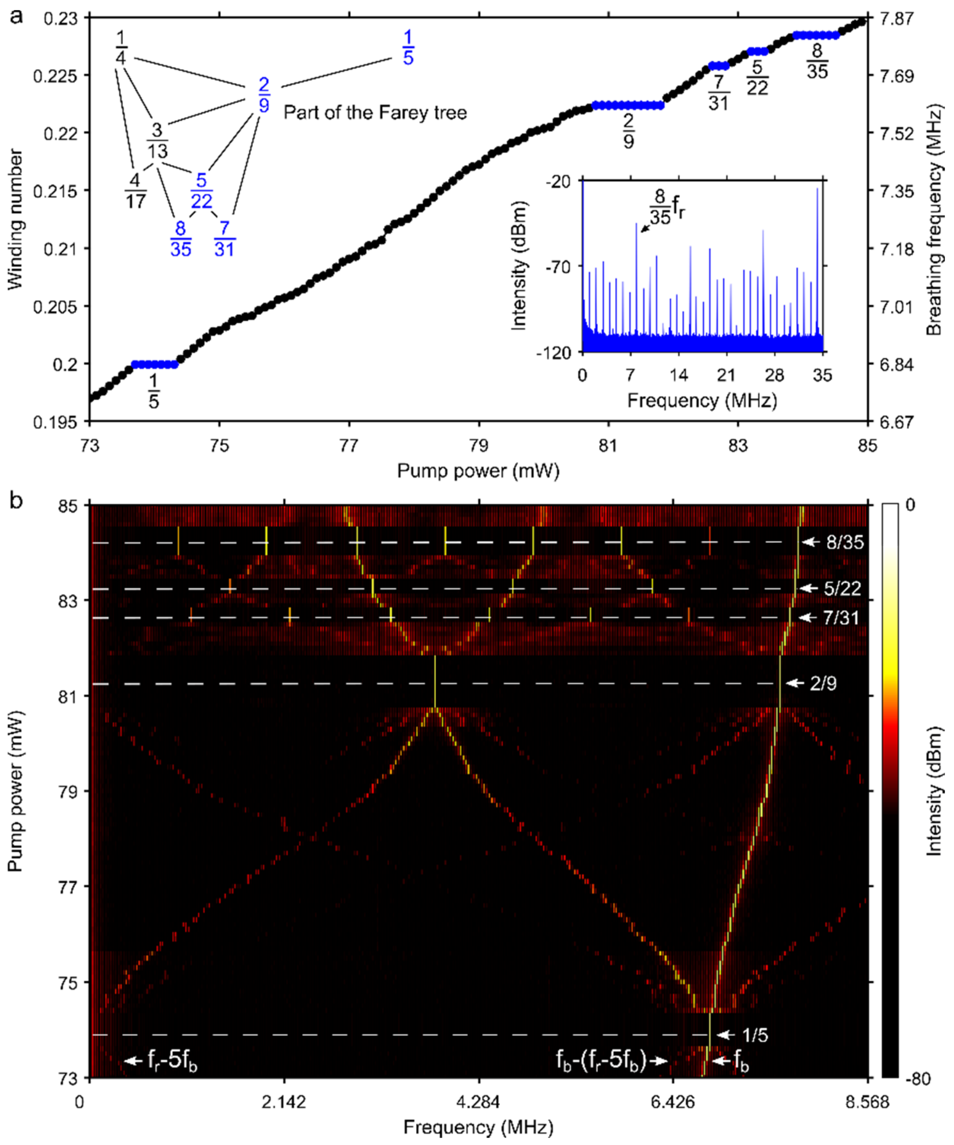

Fig. 4 RF spectra, Farey tree and devil's staircase observed by setting the laser to an initial polarisation state slightly different from that illustrated in Fig. 5 of the main body of the manuscript. (a) Measured breathing frequency (winding number) as a function of the pump power. In the insets are shown the part of the Farey tree containing the observed Farey fractions, and the RF spectrum measured with the ESA for the 8/35 frequency-locked state. The dimension of the set complementary to the stairs is calculated to be 0.807. (b) Map of spectral intensity in the space of radiofrequency and pump power, showing the build-up of rational winding numbers.

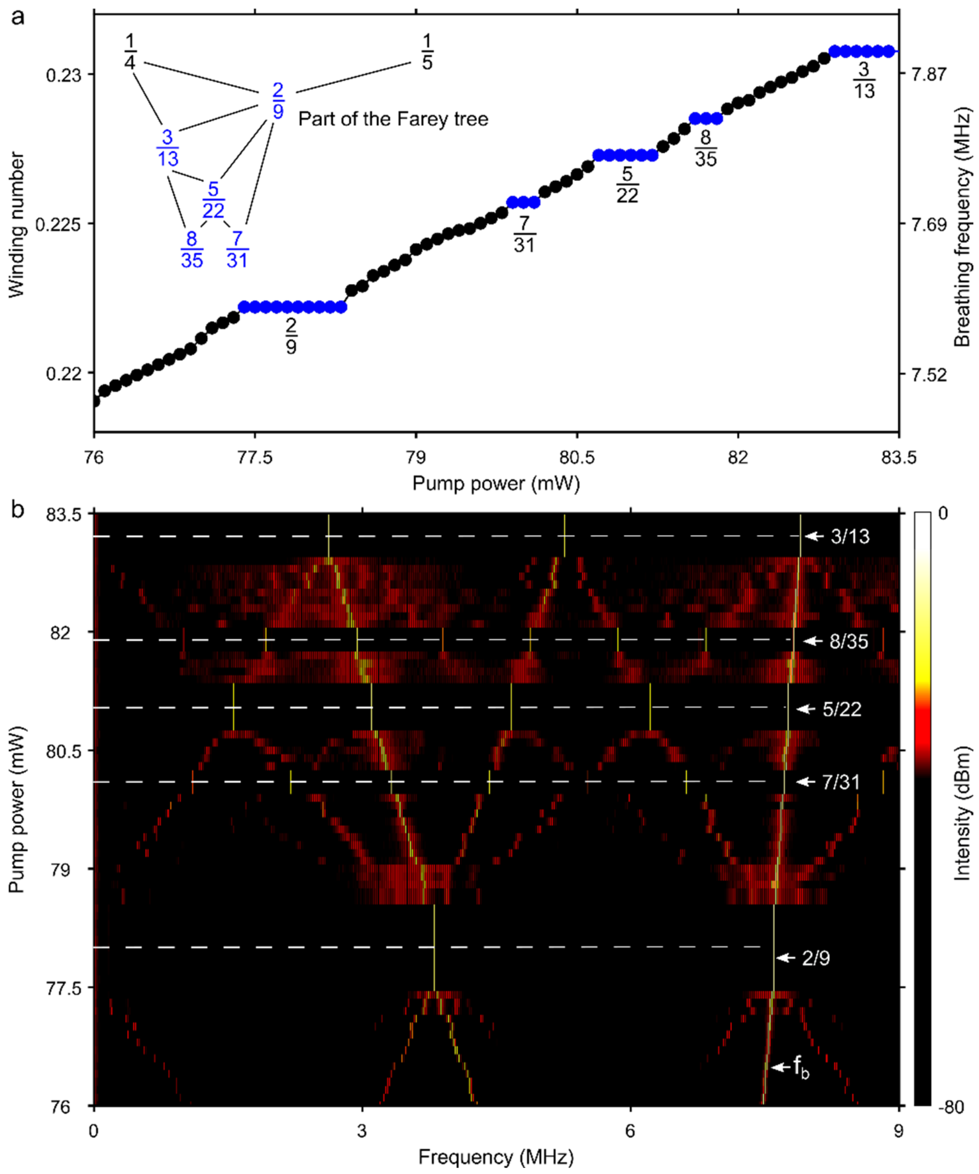

Fig. 5 Another example of a Farey tree and devil's staircase observed in the laser. (a) Measured breathing frequency (winding number) as a function of the pump power. In the inset is shown the part of the Farey tree containing the observed Farey fractions. The dimension of the set complementary to the stairs is calculated to be 0.853±0.015. (b) Map of spectral intensity in the space of radiofrequency and pump power, showing the build-up of rational winding numbers.

Table 1: Parameters of the numerical model.

Output coupler	Saturable absorber	SMF (SMF28 and Hi1060)	EDF (OFS 80)
Coupling coefficient $T_{oc} = 0.73$. This includes also other linear losses in the cavity (fibre loss, coupling from free space to fibres and splicing loss between fibres).	Unsaturated loss $q_0 = 0.3$; saturable loss $q_m = 0.4$; saturation power $P_{sat} = 40$ W	GVD coefficient β_2 = -0.0168 ps^2/m; nonlinearity coefficient γ = 0.001 $(Wm)^{-1}$; length $L = 4.3$ m. The SMF consists of SMF28 and HI1060 (from the wavelength-division multiplexer). In the model, these two fibre types for simplicity are replaced by a single SMF section with a GVD value that is the average of the two. The free space part of the laser setup, having no dispersion and nonlinearity, is negelected in the model.	$\beta_2 = 0.065$ ps^2/m; $\gamma = 0.01$ $(Wm)^{-1}$; $L = 1.25$ m; gain bandwidth = 50 nm; small-signal gain $g_0 =$ 1.8/m; gain saturation energy: $E_{sat} =$ 3670-3778 pJ (winding number f_b/f_r = 1/5); $E_{sat} =$ 4054-4071 pJ (f_b/f_r = 3/14); $E_{sat} =$ 4123-4215 pJ (f_b/f_r = 2/9).

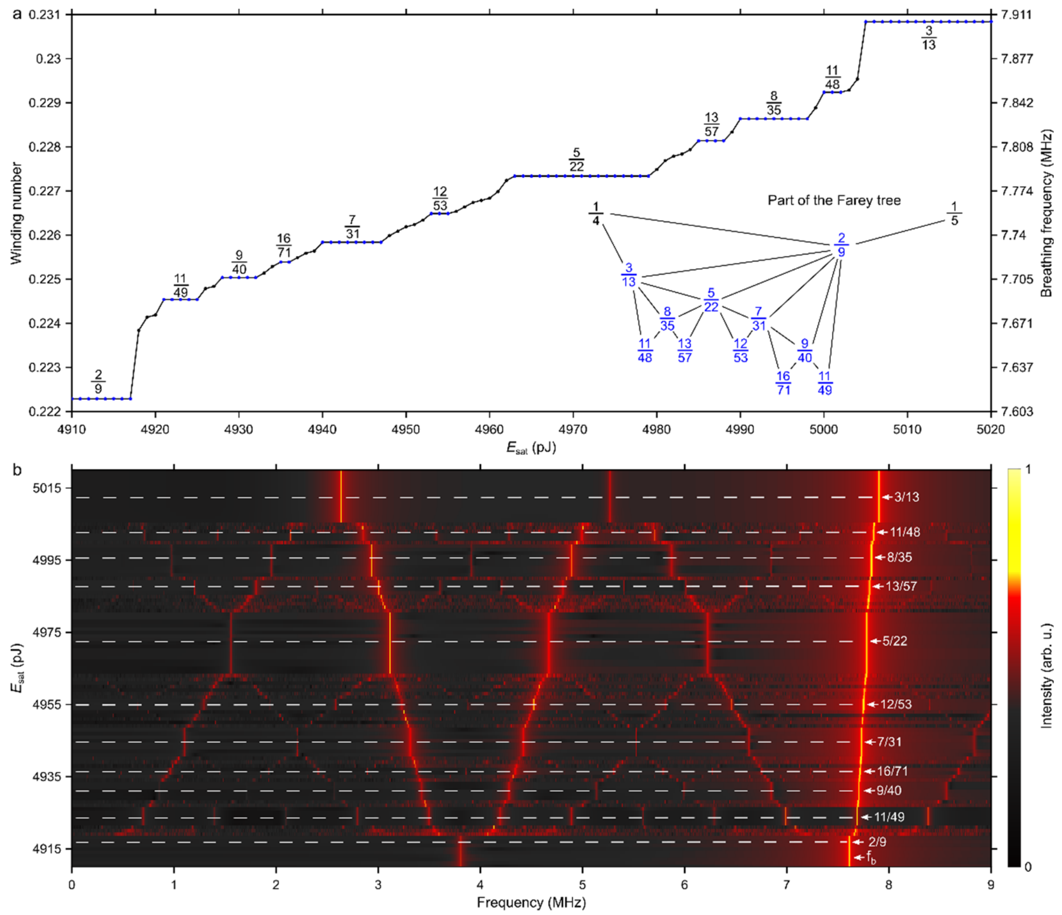

Fig. 6. Farey tree and devil's staircase observed in the numerical simulations by increasing the linear intracavity loss by 1.37% above the value used to obtain Fig. 6 in the main body of the manuscript. (a) Breathing frequency (winding number) as a function of the gain saturation energy (related to the pump power in the experiment). In the inset is shown the part of the Farey tree containing the observed Farey fractions. The dimension of the set complementary to the stairs is calculated to be 0.81±0.03. (b) Map of spectral intensity in the space of radiofrequency and gain saturation energy, showing the build-up of rational winding numbers. These numerical results relating to the experimental results shown in Fig. S5, confirm that a small change in the laser's initial polarisation state triggers Farey fractions belonging to a different part of the Farey tree. The simulations reveal more plateaux than those observed experimentally since the gain saturation energy can be varied with an arbitrarily small step in the model.
